# Verification of B-lymphocyte activating factor’s involvement in the exacerbation of insulin resistance as well as an autoimmune response in patients with nonalcoholic steatohepatitis and patients with HCV-related chronic liver disease

**DOI:** 10.1186/s13098-017-0243-z

**Published:** 2017-06-13

**Authors:** Takashi Himoto, Koji Fujita, Takako Nomura, Joji Tani, Asahiro Morishita, Hirohito Yoneyama, Reiji Haba, Tsutomu Masaki

**Affiliations:** 1 Department of Medical Technology, Kagawa Prefectural University of Health Sciences, 281-1, Hara, Mure-Cho, Takamatsu, Kagawa 761-0123 Japan; 2 Department of Gastroenterology and Neurology, Kagawa University School of Medicine, Takamatsu, Kagawa Japan; 3 Department of Diagnosis Pathology, Kagawa University School of Medicine, Takamatsu, Kagawa Japan

**Keywords:** Antinuclear antibody (ANA), B-lymphocyte activating factor (BAFF), Hepatitis C virus (HCV), Insulin resistance, Nonalcoholic steatohepatitis (NASH)

## Abstract

**Background:**

Ten to forty percent of nonalcoholic steatohepatitis (NASH) and HCV-related chronic liver disease (CLD-C) patients have antinuclear antibodies (ANAs). However, the relationship between autoimmune response and insulin resistance remains uncertain among those patients. The primary purpose of this study was to investigate whether or not ANA status was associated with the development of insulin resistance and obesity in NASH and CLD-C patients.

**Methods:**

Degrees of hepatic fibrosis and steatosis were evaluated by the classification proposed by Brunt et al. Obesity and insulin resistance were estimated by calculating body mass index and the value of homeostasis model of for assessment of insulin resistance (HOMA-IR), respectively. A revised scoring system was applied to the diagnosis of autoimmune hepatitis (AIH). Serum B-lymphocyte activating factor (BAFF) levels were determined, using an ELISA technique.

**Results:**

Ten of 25 (40%) NASH patients and 9 of 22 (41%) CLD-C patients had ANAs, though the titers were weak in most patients. Only one NASH patient met the category of “definite” AIH among the enrolled patients. Serum IgG levels were significantly higher in NASH and CLD-C patients with ANAs than in those without ANAs, and NASH and CLD-C patients with ANAs had significantly higher HOMA-IR values than those without ANAs (6.81 ± 3.36 vs. 4.00 ± 2.57, p = 0.0305, 3.01 ± 1.31 vs. 1.28 ± 0.50, p = 0.0011). CLD-C patients with ANAs had more advanced hepatic fibrosis and steatosis than those without ANAs, while ANA status was not associated with hepatic fibrosis or steatosis in NASH patients. Obesity was independent of ANA status in both subjects. Serum BAFF levels were significantly higher in CLD-C patients with ANAs than those in CLD-C patients without ANAs (1303 ± 268 vs. 714 ± 143 pg/ml, p = 0.0036). A close correlation between serum BAFF level and the HOMA-IR value was observed in CLD-C patients (r = 0.467, p = 0.0485).

**Conclusion:**

Our data suggest that NASH and CLD-C patients with ANAs have more severe insulin resistance than those without ANAs. More advanced insulin resistance deriving from excessive BAFF production may result in severe hepatic fibrosis and steatosis in CLD-C patients with ANAs.

## Background

Nonalcoholic fatty liver disease (NAFLD) is currently the most prevalent liver disease worldwide, characterized by the accumulation of triglycerides in the liver, and the absence of excessive alcohol consumption [1]. NAFLD covers a spectrum of liver diseases that range from simple steatosis called nonalcoholic fatty liver (NAFL) through nonalcoholic steatohepatitis (NASH), which is associated with hepatic inflammation and fibrosis in addition to simple steatosis [1]. Therefore, NASH is considered to be a risk factor for liver cirrhosis and hepatocellular carcinoma (HCC) [2]. It has been well established that NAFLD is a hepatic manifestation of a metabolic syndrome [3], because insulin resistance is one of the main etiological factors underlying the progression of NAFLD. Thus, the presence of NAFLD is associated with a high risk of developing type 2 diabetes mellitus (DM), dyslipidemia, and hypertension. The incidence of NAFLD will increase rapidly in the future as the frequency of obesity increases worldwide.

Hepatitis C virus (HCV) also induces a spectrum of chronic liver diseases from chronic liver disease to liver cirrhosis, and ultimately to HCC [4]. Persistent HCV infection often evokes numerous types of metabolic abnormalities, including insulin resistance, hepatic steatosis, dyslipidemia, and iron overload [5, 6]. These metabolic abnormalities are greatly involved in the development of liver damage. On the other hand, chronic HCV infection is frequently associated with autoimmune phenomena such as the emergence of non-organ-specific autoantibodies and/or concurrent extrahepatic autoimmune diseases [7].

Oxidative stress, proinflammatory cytokines, and mitochondrial dysfunction seem to be involved in the development of NASH and HCV-related chronic liver disease (CLD-C) [4, 8]. However, it remains unclear whether or not autoimmune responses are initiated during the process of disease progression. Previous studies revealed that 20–40% of patients with NAFLD [9–15] and approximately 10–50% of patients with CLD-C [16–20] have non-organ specific autoantibodies, including antinuclear antibodies (ANAs) and/or smooth muscle antibodies (SMAs) in their sera. Some investigators have reported that CLD-C patients seropositive for ANAs had significantly more advanced hepatic fibrosis than those seronegative for ANAs [21], though these findings seems to be controversial in NASH patients [12, 13]. There have been few studies to highlight the relationship between insulin resistance and autoimmune response in patients with NASH so far. Loria et al. [10] found that higher ANA titers were significantly correlated with more severe insulin resistance in patients with NAFLD.

Interestingly, a recent study using diet-induced obese mice revealed that B cells played crucial roles in insulin resistance and glucose intolerance [22]. It is well recognized that B-lymphocyte activating factor (BAFF), a member of the tumor necrosis factor-alpha (TNF-α) superfamily, is essentially involved in the survival and maturation of B cells [23]. BAFF is probably believed to be produced in adipocytes [24] as well as in macrophages, monocytes and dendritic cells, and to affect insulin receptor substrate-1 (IRS-1) in adipocyte directly [25]. Hence, we hypothesized that BAFF might facilitate not only humoral immunity but also insulin resistance in patients with NASH or CLD-C.

Furthermore, obesity has shown a strong correlation with autoimmune diseases, including autoimmune thyroiditis and type 2 DM [26, 27]. Obesity is likely to cause two distinct immunological responses: chronic inflammation through the stimulation of innate immunity and the activation of a humoral response that triggers autoantibody production [28]. We previously reported that autoimmune response was also involved in the process of hepatic steatosis in patients with chronic hepatitis C [29].

The primary purpose of the present study was to investigate the relationships between ANA status and insulin resistance, obesity, or hepatic steatosis in NASH and CLD-C patients.

## Methods

### Study population

Twenty-five patients with NASH and 22 patients with CLD-C were randomly selected from patients admitted to the Hospital of Kagawa University School of Medicine between 2003 and 2013. The pathological diagnosis of NASH was determined on the basis of the Matteoni’s classification [30].

All of the selected CLD-C patients had detectable serum HCV-RNA as determined by polymerase chain reaction (PCR) and showed histological findings compatible with chronic hepatitis or liver cirrhosis. As a comparison group, 5 cases of normal healthy control (NHC) were also assigned for this study. A revised scoring system was used to diagnose of autoimmune hepatitis (AIH) [31] among the enrolled patients.

The study protocol complied with all of the provisions of the Declaration of Helsinki. The design of this study was approved by the Ethical Committee of the Kagawa University School of Medicine, and informed consent was obtained from each individual before liver biopsy.

### Laboratory assessments

Serum alanine aminotransferase (ALT), ferritin, and immunoglobulin G (IgG) levels were measured using standard laboratory techniques. ANAs were determined by an indirect immunofluorescent method, using HEp-2 cells as a substrate. Seropositivity for ANAs was defined as titers of 1:40 or higher. Serum BAFF levels were assessed by a commercially available enzyme-linked immunosorbent assay (ELISA) kit (R&D Systems, Minneapolis, MN). These parameters in the study subjects were determined before any treatment.

Insulin resistance was evaluated based on the (HOMA-IR) value using follow equation: HOMA-IR value = Fasting insulin (µU/ml) × Fasting glucose (mg/dL)/405. Insulin resistance was defined as the values of HOMA-IR exceeding 2.5. Body mass index (BMI) was estimated as a hallmark of obesity. Obesity was defined as a BMI over 25.0 kg/m^2^, because the proportion of the population with BMI higher than 30 kg/m^2^ has been reported to be less than 2–3% in Japan [32], although the proportion of obesity in Western countries ranges from 10 to 20% [33]. These biochemical and immunological data were obtained from the enrolled patients before liver biopsy.

### Histological assessments

Liver tissue specimens were obtained by liver biopsy under ultrasound guidance, using 16-gauge needles, before treatments. The tissue samples were fixed in 10% formalin and embedded in paraffin, and then sectioned. The tissue sections were stained with hematoxylin and eosin for morphological evaluation. The degrees of hepatic fibrosis and steatosis were evaluated, using the classification proposed by Brunt et al. [34].

### Statistical analyses

Data values are represented as means ± standard deviations (SDs). The Mann–Whitney *U* test and the Bonferroni/Dunn method were applied for comparisons of 2 and 3 groups, respectively. Fisher’s exact probability test was used to compare differences in frequencies. The relationships among quantitative variables were analyzed by Pearson’s test. p values of less than 0.05 were considered significant.

## Results

### Clinical characteristics of the study subjects

The clinical characteristics of the study subjects are shown in Table 1. There were no significant differences in age, gender distribution or the severity of hepatic fibrosis between enrolled NASH and CLD-C patients. However, the degree of obesity and hepatic steatosis, and HOMA-IR value were significantly more advanced in NASH patients than in CLD-C patients. Twenty of 25 (80%) patients with NASH and 10 of 22 (45%) patients with CLD-C met the Japanese criteria for obesity. Eleven of 25 (44%) patients with NASH had grade 3 hepatic steatosis, while none of patients with CLD-C did. Seventeen of 21 (81%) patients with NASH and 6 of 22 (27%) patients with CLD-C fulfilled the category for insulin resistance. Thirteen (52%) of 25 NASH patients and 3 (14%) of 22 CLD-C patients had concurrent type 2 DM, respectively.

**Table 1:** Clinical characteristics of the enrolled patients

Clinical parameters	NASH (n = 25)	CLD-C (n = 22)	p value
Age (y.o.)	53.7 ± 13.1 (21–76)	59.1 ± 7.4 (39–69)	0.1192
Gender (male/female)	(8/17)	(10/12)	0.3824
Staging (F_1_/F_2_/F_3_/F_4_)	14/1/9/1	9/8/3/2	0.7361
Steatosis (gr0/gr1/gr2/gr3)	0/6/8/11	8/10/4/0	<0.0001
BMI	27.4 ± 4.5 (16.0–35.3)	23.8 ± 3.62 (17.0–33.5)	0.0035
HOMA-IR value	4.95 ± 3.11 (1.34–13.00)	1.99 ± 1.25 (0.53–5.74)	<0.0001
Prevalence of ANA	10 (40%)	9 (41%)	0.9999

### ANA status

**Fig. 1:**
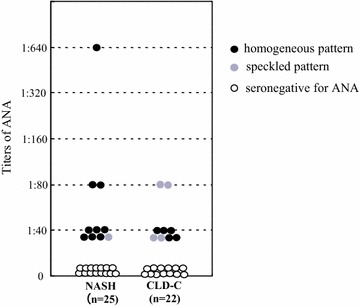
Titers and immunofluorescence patterns of ANA in patients with NASH and patients with CLD-C. *ANA* antinuclear antibody, *NASH* nonalcoholic steatohepatitis, *CLD-C* HCV-related chronic liver disease

Ten of 25 (40%) of patients with NASH and 9 of 22 (41%) patients with CLD-C had ANAs in their sera, with ANA titers ranging from 1:40 to 1:640; 7 patients with NASH and 7 patients with CLD-C showed an ANA titer of 1:40, 2 NASH patients and 2 CLD-C patients showed 1:80, and one NASH patient showed 1:640. The immunofluorescence pattern in all NASH patients but one was homogeneous, while 5 CLD-C patients showed a homogeneous pattern and four had the speckled pattern (Fig. 1).

**Fig. 2:**
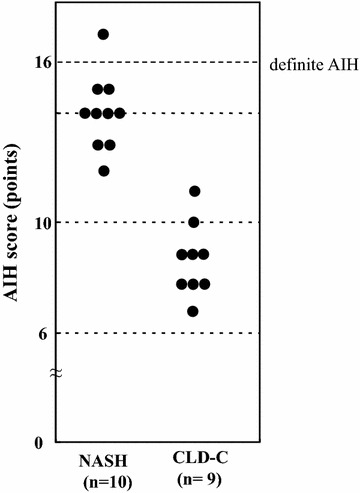
Discrimination of AIH patients in NASH patients and CLD-C patients seropositive for ANA using the revised scoring system. *AIH* autoimmune hepatitis, *NASH* nonalcoholic steatohepatitis, *CLD-C* HCV-related chronic liver disease

Next, the revised scoring system was applied to the 10 patients with NASH and 9 patients with CLD-C patients seropositive for ANAs to determine clinical diagnosis of AIH. Only one NASH patient met the criteria for ‘definite AIH’, although none of the CLD-C patients did (Fig. 2).

### Comparisons of biochemical and immunological data between NASH patients seropositive and seronegative for ANAs

Table 2 summarizes the values of biochemical, immunological, and histological parameters in NASH patients seropositive and seronegative for ANAs. Serum IgG levels in the ANA-seropositive group were significantly higher than those in the ANA-seronegative group (1883 ± 471 vs. 1292 ± 269 mg/dL, p = 0.0055). NASH patients seropositive for ANAs also had significantly higher HOMA-IR values (6.81 ± 3.46 vs. 4.00 ± 2.57, p = 0.0305), indicating that autoimmune response is involved in the progression of insulin resistance. Interestingly, serum ferritin levels were significantly higher in NASH patients with ANAs than in those without ANAs (518 ± 270 vs. 229 ± 97 ng/ml, p = 0.0041). However, no significant differences in serum ALT levels or BMI were found between ANA-seropositive and ANA-seronegative NASH patients.

**Table 2:** Comparisons of clinical parameters between NASH patients seropositive and seronegative for ANAs

	ANA-positive (n = 10)	ANA-positive (n = 15)	p value
Age (y.o.)	55.0 ± 6.3	52.9 ± 16.4	0.9115
Gender (male/female)	4/6	4/11	0.7794
BMI	28.3 ± 5.0	26.9 ± 4.3	0.6773
HOMA-IR value	6.81 ± 3.46 (n = 7)	4.00 ± 2.57 (n = 14)	0.0305
ALT (IU/L)	108 ± 82	135 ± 75	0.2018
IgG (mg/dL)	1833 ± 471	1292 ± 269	0.0055
Ferritin (ng/ml)	518 ± 270	229 ± 97	0.0041
Hepatic fibrosis (F_0_/F_1_/F_2_/F_3_/F_4_)	1/2/1/6/0	0/8/2/4/1	0.4055
Hepatic steatosis (grade 0/1/2)	3/2/5	6/5/4	0.3458

### Comparisons of biochemical and immunological data between the groups of CLD-C patients seropositive and seronegative for ANAs

CLD-C patients with ANA had significantly higher serum ALT, IgG and ferritin levels, and HOMA-IR values than CLD patients without ANAs. However, BMI was found to be independent of ANA status in patients with CLD-C (Table 3).

**Table 3:** Comparisons of clinical parameters between CLD-C patients seropositive and seronegative for ANAs

	ANA-positive (n = 9)	ANA-positive (n = 13)	p value
Age (y.o.)	62.2 ± 5.9	56.9 ± 7.7	0.0883
Gender (male/female)	3/6	7/6	0.4149
BMI	24.5 ± 4.4	23.3 ± 3.1	0.4905
HOMA-IR value	3.01 ± 1.31	1.28 ± 0.50	0.0011
ALT (IU/L)	129 ± 90	38 ± 22	0.0024
IgG (mg/dL)	2282 ± 336	1467 ± 244	0.0008
Ferritin (ng/ml)	450 ± 405	141 ± 87	0.0056
Hepatic fibrosis (F_0_/F_1_/F_2_/F_3_/F_4_)	0/5/2/2	9/3/1/0	0.0014
Hepatic steatosis (grade 0/1/2)	2/3/4	6/7/0	0.0430

### Comparisons of histological findings between the groups of NASH patients seropositive and seronegative for ANAs

The severity of hepatic fibrosis was compared between the groups of NASH patients with and without ANAs. As shown in Table 2, ANA status was independent of hepatic fibrosis, steatosis, and BMI in the enrolled NASH patients. In contrast, the degrees of hepatic fibrosis and steatosis were significantly more severe in CLD-C patients with ANAs, compared to those in CLD-C patients without ANAs (Table 3). BMI was not related to ANA status in CLD-C patients.

### Correlation between serum BAFF levels and other clinical parameters

**Fig. 3:**
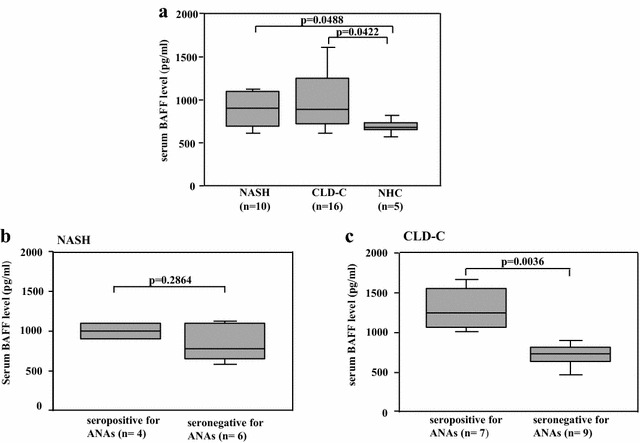
**a** Comparison of serum BAFF levels in all subjects. **b** Comparison of serum BAFF levels between NASH patients seropositive and seronegative for ANAs. **c** Comparison of serum BAFF levels between CLD-C patients seropositive and seronegative for ANAs. The *boxes* represent the values within 25th and 75th percentiles. The *horizontal bars* represent the medians. *BAFF* B-lymphocyte activating factor, *NASH* nonalcoholic steatohepatitis, *NHC* normal healthy control, *CLD-C* HCV-related chronic liver disease

**Fig. 4:**
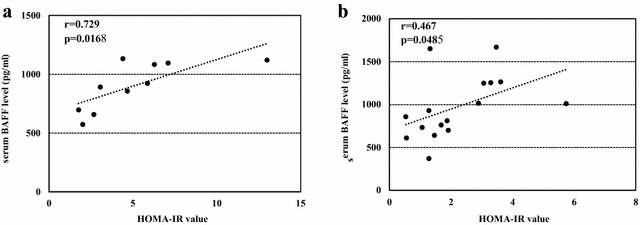
Relationship between serum BAFF levels and insulin resistance in patients with NASH (**a**) and patients with CLD-C (**b**). *BAFF* B-lymphocyte activating factor, *NASH* nonalcoholic steatohepatitis, *CLD-C* HCV-related chronic liver disease, *HOMA-IR* homeostasis model for assessment of insulin resistance

**Fig. 5:**
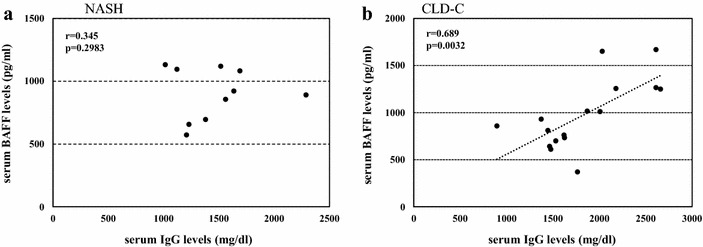
Relationship between serum BAFF and IgG levels in patients with NASH (**a**) and patients with CLD-C (**b**). *BAFF* B-lymphocyte activating factor, *NASH* nonalcoholic steatohepatitis, *CLD-C* HCV-related chronic liver disease

Serum BAFF levels were determined in 10 of 25 NASH patients, 16 of 22 CLD-C patients and 5 cases of NHCs to investigate the relationship between autoimmune response and insulin resistance. The serum BAFF levels in NASH and CLD-C patients were significantly higher than those in NHCs (903 ± 206, 972 ± 366 vs. 690 ± 88 pg/ml, Fig. 3a). As shown in Fig. 4a, serum BAFF levels roughly correlated with HOMA-IR values (r = 0.729, p = 0.0168) in patients with NASH. ANA-seropositive NASH patients tended to show higher serum BAFF levels, compared to those in ANA-seronegative NASH patients (1004 ± 114 vs. 835 ± 235 pg/ml, p = 0.2864, Fig. 3b). However, serum BAFF levels were found to be independent of serum ALT (r = 0.249, p = 0.4874), IgG (r = 0.345, p = 0.2983, Fig. 5a) and ferritin (r = 0.476, p = 0.1950) in those patients.

Similarly, serum BAFF levels were significantly higher in CLD-patients with ANAs than in those without ANAs (1303 ± 268 vs. 714 ± 143 pg/ml, p = 0.0036, Fig. 3c). Serum BAFF levels also showed a significant correlation with HOMA-IR value in CLD-C patients (r = 0.467, p = 0.0485, Fig. 4b). The serum BAFF levels were also linked to the serum IgG levels in patients with CLD-C (r = 0.689, p = 0.0032, Fig. 5b). However, no significant relationship was found between serum BAFF and ALT (r = 0.402, p = 0.1229) or ferritin (r = 0.342, p = 0.1950) levels in patients with CLD-C.

## Discussion

Our results support our hypothesis that BAFF is involved in the progression of insulin resistance in NASH or CLD-C patients. Moreover, we confirmed that serum BAFF levels were associated with ANA status in CLD-C patients. BAFF is likely to play a crucial role in HCV-associated B cell proliferation [35]. However, to the best of our knowledge, this is the first report to focus primarily on the relationship between insulin resistance and autoimmune response via BAFF overproduction in CLD-C patients.

Here, we propose a putative mechanism by which BAFF participates in the emergence of ANAs and the enhancement of insulin resistance as follows: overproduction of BAFF in adipocytes leads to the polyclonal activation of B cells and subsequent initiation of ANA production. Simultaneously, excessive BAFF directly affects the down-regulation of IRS-1 and eventually exacerbates insulin resistance.

In the present study, the prevalence of ANAs in patients with NASH or CLD-C was approximately similar to that previously reported [10–19], and ANA titers in the sera of most NASH or CLD-C patients were as weak as those previously elucidated [10–14, 16–18]. The present data demonstrated that CLD-C patients with ANAs had more advanced hepatic fibrosis and steatosis than CLD-C patients without ANAs, while ANA status was not associated with the progression of hepatic fibrosis or steatosis in NASH patients. It has been widely recognized that hepatic fibrosis and steatosis were closely linked to insulin resistance in CLD-C patients [37, 38], and that CLD-C patients with ANAs showed more advanced hepatic fibrosis than those without ANAs [21]. Therefore, greater BAFF production results in more severe insulin resistance, possibly leading to more advanced hepatic fibrosis and steatosis in CLD-C patients with ANAs. However, the elevation of serum BAFF level was somewhat mild in NASH patients with ANA, compared to that in CLD-C patients with ANAs. Mild elevation of BAFF in NASH patients with ANAs was not associated with any significant difference in the degrees of hepatic fibrosis or steatosis between ANA-seropositive and ANA-seronegative NASH patients.

The present study revealed that BMI was associated with neither ANA status nor serum IgG level (data not shown) in NASH or CLD-C patients, suggesting that obesity might not be responsible for the development of autoimmunity in the diseases under consideration. Apoptosis inhibitor of macrophage (AIM), which induces a class-switch of macrophages, and consequently leads to chronic inflammation, appears to participate in obesity-associated autoimmune responses. Taking our results into consideration, AIM is unlikely to be involved in the development of NASH or CLD-C [36].

It was of interest that serum ferritin levels proved to be significantly higher in NASH patients with ANAs than in NASH patients without ANAs. Similarly, CLD-C patients seropositive for ANAs had significantly higher serum ferritin levels than CLD-C patients seronegative for ANAs, indicating that an autoimmune response was involved in iron storage in the liver of these patients. We often experienced CLD-C patients whose serum ferritin levels are transiently elevated during pegylated interferon (PEG-IFN)-based treatment [39]. The elevation of serum ferritin level in patients with CLD-C patients may account for the activation of Kupffer cells by the administration of PEG-IFN [40]. In addition, an elevation of serum ferritin level in patients with systemic lupus erythematosus (SLE) may imply the active stage of the disease [41], suggesting the possibility that ferritin may be a novel serological autoimmune hallmark. Unfortunately, the present study did not show the significant correlations between serum BAFF and ferritin levels in NASH and CLD-C patients.

There were several limitations in the present study. First, our study was limited by a small sample size, although we acquired the valuable results in the present study. A larger-scale cohort study is required to confirm the present results. Second, we were unable to identify the origin to facilitate the synthesis of BAFF, although we speculated that adipocytes are a good candidate. The extent and location of BAFF receptor were not investigated in this study, either. Third, laboratory autoimmune parameters other than ANAs were not determined in the enrolled patients. Therefore, the titers of autoantibodies other than ANAs might be associated with serum BAFF levels in patients with NASH.

## Conclusion

We concluded that an increase in BAFF synthesis may evoke exacerbation of insulin resistance as well as an autoimmune response in NASH and CLD-C patients. The exacerbation of insulin resistance by way of BAFF overproduction may result in the progression of hepatic fibrosis and steatosis in ANA-seropositive CLD-C patients.

## Abbreviations

AIH: autoimmune hepattis
AIM: apoptosis inhibitor of macrophage
ALT: alanine aminotransferase
ANA: antinuclear antibody
BAFF: B-lymphocye activating factor
BMI: body mass index
CLD-C: HCV-related chronic liver disease
DM: diabetes mellitus
HCC: hepatocellular carcinoma
HCV: hepatitis C virus
HOMA-IR: homeostasis model for assessment of insulin resistance
IgG: immunoglobulin G
NAFLD: nonalcoholic fatty liver disease
NASH: nonalcoholic steatohepatitis
NHC: normal healthy control
